# Impact of negative symptom reduction on employment and vocational activity outcomes following cognitive remediation therapy in schizophrenia: A 1‐year follow‐up study

**DOI:** 10.1002/pcn5.70117

**Published:** 2025-05-07

**Authors:** Yasuhisa Nakamura, Keito Shimada, Reiko Miyamoto, Akihiro Koreki, Sachiko Anamizu, Masaru Mimura

**Affiliations:** ^1^ Department of Rehabilitation, Faculty of Health Sciences Nihon Fukushi University Aichi Japan; ^2^ Kariya Hospital Psychiatry Day Care Aichi Japan; ^3^ Division of Occupational Therapy, Faculty of Health Sciences Tokyo Metropolitan University Tokyo Japan; ^4^ Department of Neuropsychiatry Keio University School of Medicine Minato Japan; ^5^ Department of Psychiatry NHO Tochigi Medical Center Tochigi Japan

**Keywords:** cognitive remediation therapy, employment transition, occupational outcomes, psychiatric daycare, schizophrenia

## Abstract

**Aim:**

Cognitive remediation therapy (CRT) improves cognitive function and enhances vocational outcomes in patients with schizophrenia. However, factors that facilitate employment or vocational activity transition following CRT remain unclear. This study aimed to identify such factors by evaluating the effects of CRT on psychiatric symptoms, cognitive function, divergent thinking, and daily living skills.

**Methods:**

Twenty‐one patients with schizophrenia underwent CRT using the Japanese Cognitive Rehabilitation Programme for Schizophrenia. The program included 24 sessions over 3 months, conducted twice weekly in small groups. Each session combined computerized cognitive training and a bridging session to support real‐life application. Pre‐ and post‐CRT assessments included the Global Assessment of Functioning (GAF), Positive and Negative Syndrome Scale (PANSS), Brief Assessment of Cognition in Schizophrenia (BACS), modified Tinkertoy Test (m‐TTT), Idea Fluency Test (IFT), Design Fluency Test (DFT), and Life Skills Profile (LSP). One year after CRT, participants were classified as engaged in employment or vocational activity or not, and group comparisons identified factors linked to vocational transition.

**Results:**

Pre‐ and postintervention comparisons showed significant improvements in GAF, PANSS, BACS, m‐TTT, IFT, DFT, and LSP scores. In a between‐group comparison, those who transitioned to employment or vocational activity (*n* = 9) exhibited significantly greater reductions in PANSS‐negative symptoms than those who did not (*n* = 12, P = 0.03).

**Conclusion:**

This study suggests an association between reduced negative symptoms and vocational engagement after CRT. Further research with larger samples is needed to clarify this relationship and enhance outcomes through targeted interventions.

## Graphical abstract

This study investigates the impact of cognitive remediation therapy (CRT) on employment outcomes in patients with schizophrenia. Findings highlight that reductions in negative symptoms, facilitated by CRT, play a critical role in successful employment transitions. The results underscore the importance of holistic rehabilitation approaches to support real‐world functional recovery.

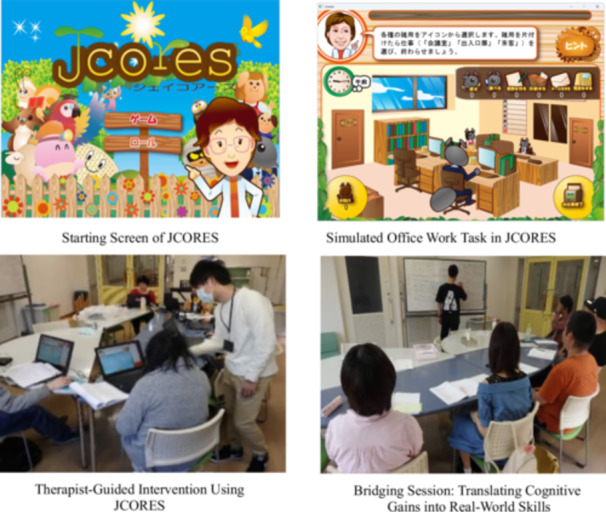

## INTRODUCTION

Schizophrenia is a chronic mental disorder that impairs cognitive and functional capacities, creating significant barriers to vocational participation.^1^, ^2^, ^3^ Affecting ∼1% of the global population, it poses a major public health concern.^4^ Cognitive deficits, particularly in memory, attention, and executive functioning, hinder vocational integration and contribute to long‐term unemployment, social exclusion, and economic hardship.^5^, ^6^, ^7^

Cognitive remediation therapy (CRT) has emerged as a promising intervention for addressing these impairments.^8^ By targeting core cognitive domains—such as working memory, attention, and executive function—CRT aims to improve cognitive performance and support broader functional outcomes, including enhanced vocational engagement.^9^, ^10^ It is now regarded as a key component of comprehensive rehabilitation programs for individuals with schizophrenia.^11^, ^12^, ^13^

Amado et al. reported that personalized CRT led to improved long‐term vocational outcomes, with 57.6% of participants achieving some form of employment 2–9 years after the intervention, including 36.4% in competitive jobs.^14^ These findings suggest that CRT may have durable effects on vocational participation by enhancing executive function and working memory, which are essential for job performance.^15^, ^16^

Beyond cognitive improvements, CRT may also support broader functional recovery. Longitudinal studies have shown sustained cognitive gains, improved community functioning, and delayed relapse following CRT.^17^, ^18^ These effects are likely to contribute indirectly to vocational engagement.

However, it remains unclear which specific factors mediate successful vocational transitions after CRT. A certain period may be needed for CRT‐induced cognitive gains to translate into behavioral change and real‐world skills necessary for work participation. Identifying these mechanisms is critical for optimizing CRT's vocational impact.

A systematic review reported that vocational participation among individuals with schizophrenia varies from 4% to 50%, influenced by factors such as age,^19^ premorbid intelligence quotient (IQ),^20^ medication dosage,^21^ motivation for CRT,^22^ and cognitive gains.^23^ However, few studies have directly compared individuals who transitioned to vocational activity after CRT with those who did not.

In this study, we examined the effects of CRT on psychiatric symptoms, cognitive function, divergent thinking, and life skills in individuals with schizophrenia. We also investigated factors distinguishing those who transitioned to employment or vocational activity within 1 year of CRT from those who did not, aiming to inform more effective vocational rehabilitation strategies.

## METHODS

### Study design

Employment transition was defined as engaging in structured vocational activity for at least 3 months, including welfare‐based employment (e.g., non‐Type A support programs), supported employment, part‐time employment, or regular employment.

While some forms of welfare‐based employment may not involve formal labor contracts, they are institutionally recognized within Japan's national disability support system as valid employment pathways, therefore transitions from psychiatric daycare to these settings were categorized as employment transitions in this study.

For clarity, in this study, “employment” was defined as engagement in work under a formal labor contract, including part‐time or disability employment that meets legal wage and insurance requirements.

In contrast, “vocational activity” referred to structured work‐related programs such as Type B continuous employment support, which are not classified as formal employment under national labor law. However, they represent meaningful involvement in vocational preparation and social participation.

Participants who engaged in either formal employment or vocational activity for at least 3 months during the 12‐month follow‐up were classified as having achieved vocational transition.

### Participants

The study initially involved 26 individuals who were referred for participation. The inclusion criteria required participants to have a diagnosis of schizophrenia confirmed by a psychiatrist according to the Diagnostic and Statistical Manual of Mental Disorders, Fifth Edition (DSM–5). All participants received outpatient pharmacotherapy and had psychiatric symptoms that were stable enough to permit community living. During CRT, all participants were attending a psychiatric daycare program and were not receiving alternative employment support services.

The exclusion criteria included severe neurological or psychiatric conditions, a history of alcohol dependence or substance abuse, or an inability to continue participation. Among the 23 participants who completed the study, seven individuals (30.4%) took anticholinergic medications and nine (39.1%) took benzodiazepines during CRT. These details are provided in Table 1.

**Table 1:** Baseline characteristics of the study participants.

Variable	Total (*n* = 23)	Completers (*n* = 21)	Dropouts (*n* = 2)	*p* value
Age (years)	40.1 ± 6.7	40.7 ± 6.7	34.0 ± 4.2	0.55
Sex (male/female)	16/7	14/7	2/0	1.00
Education	13.2 ± 1.7	13.1 ± 1.7	13.5 ± 2.1	0.87
Duration of illness	17.4 ± 8.0	17.6 ± 8.2	16.0 ± 5.7	0.71
Living arrangements				
Living with family	17	16	1	
Living alone	6	5	1	0.46
Past employment experience				
Yes (≥3 months of employment)	19	17	2	
No (<3 months or never)	4	4	0	1.00
Total duration of employment (months)	74.1 ± 95.6	80.3 ± 99.4	21.0 ± 4.2	0.77
Chlorpromazine equivalent (mg)	518.7 ± 276.4	515.7 ± 289.2	375.0 ± 318.9	0.57
Anticholinergic use	7	6	1	
No anticholinergic use	16	15	1	0.56
Benzodiazepine use	9	8	1	
No benzodiazepine use	14	13	1	1.00
JART IQ	99.0 ± 9.4	98.8 ± 9.8	102 ± 0	0.77
BACS composite score	40.4 ± 12.3	39.9 ± 12.5	46.0 ± 11.3	0.71
PANSS positive syndrome	19.8 ± 4.2	20.0 ± 4.3	17.0 ± 0	0.39
PANSS negative syndrome	20.0 ± 3.4	21.1 ± 3.3	18.0 ± 0	0.40
PANSS general psychopathology	41.2 ± 6.0	41.5 ± 6.2	40.5 ± 2.1	0.52

Abbreviations: BACS, Brief Assessment of Cognition in Schizophrenia; IQ, intelligence quotient; JART, Japanese Adult Reading Test; PANSS, Positive and Negative Syndrome Scale.

### Intervention

CRT was implemented using the Japanese Cognitive Rehabilitation Program for Schizophrenia (JCORES) software, a structured cognitive training program that targets memory, attention, and executive functions. The intervention included 36 sessions over 3 months, including two computerized cognitive training sessions followed by one bridging session per set. This structure was repeated 24 times for cognitive training (24 sessions) and 12 times for bridging sessions (12 sessions). Each session lasted ∼90 min: 60 min of computer‐based training and 30 min of a bridging session aimed at applying cognitive skills to real‐life situations.

**Figure 1:**
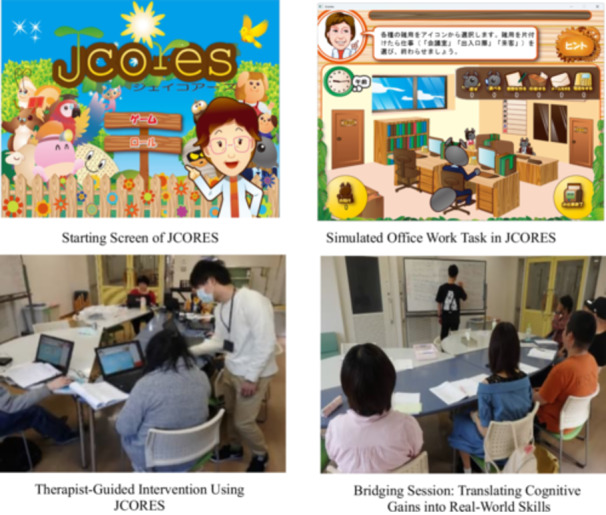
Illustrative overview of the Japanese Cognitive Rehabilitation Program for Schizophrenia (JCORES)‐based cognitive remediation therapy and bridging sessions in practice.

An overview of the JCORES‐based CRT and bridging sessions is presented in Figure 1.

### JCORES‐based cognitive training

The JCORES program provides a range of computerized exercises targeting five core cognitive domains: attention, psychomotor speed, learning, memory, and executive function.

Each cognitive domain includes three to seven types of games with five difficulty levels, allowing participants to adjust tasks based on their strengths, weaknesses, and preferences. The program follows an adaptive hierarchical design, starting with fundamental cognitive processes (e.g., attention, psychomotor speed) and progressing to complex skills such as verbal memory, reasoning, and problem‐solving. The final phase involves training in real‐life scenarios with increased contextual relevance to work and daily life. Additionally, auditory training is incorporated to supplement visual tasks because accurate speech perception is essential for effective communication in occupational settings. Previous studies have demonstrated that JCORES significantly improves verbal memory and reduces overall psychopathology in patients with schizophrenia.^24^ Furthermore, it has been reported that the intervention induces microstructural changes in cerebellar regions involved in cognitive functions.^25^

### Intervention setting

Although all participants were attending a psychiatric daycare program during CRT, the facility did not provide structured vocational support. Instead, participants received only casework services based on their needs and preferences. These casework services included goal‐setting support, personal counseling, and guidance on social and daily living skills; however, they did not involve systematic job training or employment placement. As a result, CRT was implemented as a stand‐alone cognitive rehabilitation intervention within the daycare setting.

### Implementation and role of therapists

Two occupational therapists with over 15 years of clinical experience conducted the intervention, both of whom had completed specialized CRT training. Their role was to monitor participants' progress, provide individualized feedback, encourage engagement, and guide cognitive strategy development. To ensure objectivity, the therapists conducting the intervention were different from those performing assessments. Sessions were conducted in small groups of approximately five participants, allowing for peer support and collaborative learning.

### Bridging sessions for real‐life application

The 30‐min bridging sessions were structured group discussions designed to connect cognitive training to real‐world employment and daily life challenges. In the early sessions, participants reflected on tasks they found easy or difficult and discussed cognitive strategies to improve performance. In mid‐to‐late sessions, discussions focused on problem‐solving strategies for work‐related scenarios, including preparing for employment, understanding the role of cognition in job tasks, and developing compensatory strategies to overcome workplace difficulties. These discussions helped participants set personal goals for community reintegration and track their progress. By integrating structured cognitive training with guided discussions, the program aimed to enhance cognitive functions and facilitate their transfer to occupational settings.

### Assessments

To compare neurocognitive function before and after the JCORES and bridging sessions, we administered the Brief Assessment of Cognition in Schizophrenia (BACS). Additionally, to evaluate divergent thinking, we administered the Idea Fluency Test (IFT), Design Fluency Test (DFT), and modified Tinkertoy Test (m‐TTT). The IFT measures verbal fluency, DFT assesses visual‐spatial ideation, and m‐TTT evaluates problem‐solving flexibility. We also assessed psychiatric symptoms, functioning, and life skills using the Positive and Negative Syndrome Scale (PANSS), Global Assessment of Functioning (GAF), and Life Skills Profile (LSP), respectively.

All assessments were conducted at two time points: immediately before the start of the CRT program (pre‐intervention) and immediately after the completion of the 3‐month CRT program (post‐intervention). Employment status was evaluated 1 year after the CRT intervention ended. The 12‐month follow‐up period started after the intervention, excluding the 3‐month CRT implementation period.

### Neuropsychological assessments

#### Brief Assessment of Cognition in Schizophrenia

The BACS assesses multiple aspects of cognitive functioning in schizophrenia and includes six measures: verbal memory, working memory, motor speed, verbal fluency, attention, and executive functioning. Each of the six measures was standardized by creating *T*‐scores. The composite score was calculated by averaging all *T*‐scores of the six measures.^26^ These assessments were primarily conducted by the lead author, with efforts to maintain consistency across all evaluations. Although an independent review of the results was not systematically performed for all cases, steps were taken to ensure that the assessments followed standardized protocols.

To minimize practice effects associated with repeated assessments, we used two alternative versions of the BACS—A‐part and B‐part—administered at different time points. These versions are designed to be equivalent in difficulty while differing in content, reducing the likelihood of participants improving their scores due to familiarity with the test materials. The BACS has been validated for its reliability and effectiveness in assessing cognitive impairments in schizophrenia.^27^

### Divergent thinking

#### Idea Fluency Test

In the IFT, the examiner asked participants to think of all possible uses of an empty tin within 5 min. Responses were classified into three categories: task‐dependent (Idea Td), task‐modified (Idea Tm), and task‐independent (Idea Ti).^28^ The lead author, supported by trained research staff, conducted these assessments. Although the lead author primarily categorized the responses, the classification criteria were carefully applied consistently.

#### Design Fluency Test

In the DFT, participants were asked to produce as many drawings as possible for one set of four dots within 3 min. The examiner classified the drawings into three categories: task‐dependent (Design Td), task‐modified (Design Tm), and task‐independent (Design Ti).^28^ The lead author performed these assessments and endeavored to apply the classification criteria uniformly, although the review process did not involve additional independent raters.

#### modified Tinkertoy Test

The m‐TTT was used to assess divergent thinking. Participants were asked to construct any structure they desired using 50 pieces from a Tinkertoy set; the only instruction was to “make whatever they wanted.” The lead author conducted the test and scored the complexity and creativity. Although an independent evaluator did not cross‐verify the scoring process, the lead author adhered to an established scoring system.

The m‐TTT has been widely used in psychiatric and neuropsychological research as a measure of executive function and creativity in patients with schizophrenia. The test was originally conceptualized as an executive function assessment,^29^, ^30^ and its criterion‐related validity has been corroborated with verbal and nonverbal divergent thinking scores.^31^ Additionally, m‐TTT performance is associated with daily living skills in patients with schizophrenia.^32^

### Psychiatric symptoms

#### Positive and Negative Syndrome Scale

The PANSS was used to obtain scores on the positive, negative, and general psychopathology subscales. The participants' primary psychiatrists conducted the measurements for this scale. It is a widely validated tool for measuring symptom severity in schizophrenia.^33^, ^34^

### Functional assessment

#### Global Assessment of Functioning

The GAF was used to assess the psychological, social, and occupational functioning of patients with schizophrenia.^35^ The measurements for this scale were conducted by the participants' primary psychiatrists, who were trained and experienced in using it. Studies have shown that GAF scores are reliable indicators of functional impairment in patients with schizophrenia.^36^

### Community living skills

#### Life Skills Profile

The LSP, developed by Rosen et al., measures disabilities in daily life and community functioning in patients with chronic mental disorders. The reliability and validity of this scale have been previously confirmed.^37^, ^38^, ^39^

The LSP consists of five subscales comprising 39 items: self‐care, non‐turbulence, socialization, communication, and responsibility. Each item is rated on a scale from 1–4, with higher scores indicating better functional outcomes. Family members, psychiatric professionals, and case workers can serve as informants for this assessment.

The measurements were conducted by daycare staff who were not involved in the intervention, ensuring that the assessments were unbiased and reflective of the participants' true abilities.

### Statistical analysis

Pre‐ and postintervention comparisons were conducted using paired *t*‐tests to assess the effects of CRT. Effect sizes (Cohen's *d*) were calculated to evaluate the magnitude of change.

Participants were then categorized into two groups based on their employment status 1 year after the intervention: those who transitioned to employment and those who did not. Group comparisons of demographic characteristics, baseline scores, and changes in scores were conducted using independent *t*‐tests, *χ* ^2^ tests, or Fisher's exact tests, as appropriate.

Logistic regression analysis was performed using employment status (employed vs. not employed) as the dependent variable to identify predictors of employment transition.

Independent variables were selected based on a large effect size (Cohen's *d* ≥ 0.8) observed in group comparisons of change scores. This threshold was applied to focus on variables with substantial group differences while minimizing the risk of overfitting due to the small sample size (*n* = 21). The regression model used the likelihood‐ratio forward stepwise method for variable entry.

All statistical analyses were conducted using IBM SPSS Statistics, version 21.0 for Windows. A two‐tailed P‐value of <0.05 was considered statistically significant.

## RESULTS

### Participants

**Figure 2:**
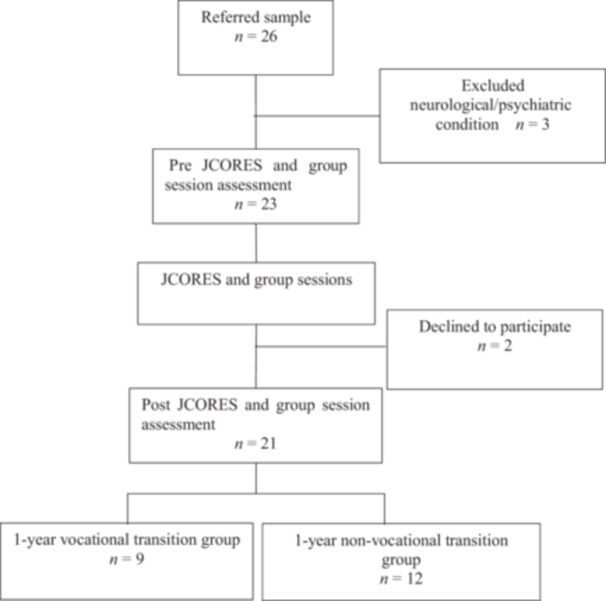
Study flowchart illustrating vocational transition status 1 year after cognitive remediation therapy. JCORES, Japanese Cognitive Rehabilitation Program for Schizophrenia.

The study initially included 26 participants; 23 were assessed before the JCORES and bridging sessions. Three participants were excluded before the assessment due to neurological or psychiatric conditions. During the intervention, two participants dropped out. There were no significant differences in baseline characteristics or assessment scores between completers and dropouts (Table 1). The study flowchart is presented in Figure 2, outlining participant recruitment, intervention, and follow‐up.

One year after the intervention, participants were categorized as those who transitioned to employment (*n* = 9) and those who did not (*n* = 12). The analysis included data from these 21 participants (Figure 2).

### Employment outcomes

Among the 21 participants who underwent CRT, nine individuals (42.8%) transitioned from psychiatric daycare to either formal employment or vocational activity within 1 year after the intervention.

Of these, four participants (19.0%) obtained formal employment based on labor contracts: two through part‐time employment and two through disability employment programs.

The remaining five participants (23.8%) engaged in vocational activity, specifically welfare‐based work settings such as Type B continuous employment support services, which are not legally classified as employment but are recognized as structured prevocational engagement under Japan's disability support system.

For context, we also examined outcomes among ∼180 individuals in the same psychiatric daycare who did not receive CRT. During the same 1‐year period, only one obtained formal employment (0.6%) and two engaged in vocational activity (1.1%).

The average time to transition (employment or vocational activity) following CRT was 191.1 ± 106.3 days. No significant correlation was observed between transition timing and clinical or demographic variables.

While differences in participant characteristics may have influenced outcomes, these findings suggest that CRT may support vocational transition, including both formal employment and preparatory vocational engagement.

### Comparison of pre‐ and postintervention with JCORES and bridging sessions

A total of 24 interventions were conducted using computer‐based cognitive training with JCORES and group sessions. Participants attended 20–24 sessions, with an average attendance of 23.0 ± 1.2 sessions. The analysis included 21 participants with a mean age of 40.7 ± 6.6 years, of whom 14 were male and seven were female. Participants had a mean of 12.9 ± 1.9 years of education and a mean illness duration of 17.6 ± 8.2 years. The chlorpromazine (CP) equivalent dose was 515.7 ± 289.2 mg and the mean Japanese Adult Reading Test (JART) IQ score was 98.8 ± 9.8.

The scores of the evaluation items before and after the intervention are presented in Table 2. Significant differences were observed in the following evaluation items pre‐ and post‐intervention: BACS verbal memory (P = 0.01, effect size = 0.71, 95% confidence interval [CI] 0.22–1.18), working memory (P = 0.01, effect size 0.85, 95% CI 0.34–1.35), motorspeed (P = 0.01, effect size = 1.06, 95% CI 0.49–1.56), verbal fluency (P = 0.01, effect size = 0.90, 95% CI 0.38–1.40), composite score (P = 0.01, effect size = 1.38, 95% CI 0.97–1.00), Idea Tm (P = 0.04, effect size = 0.58, 95% CI 0.07–0.85), m‐TTT total score (P = 0.01, effect size = 0.75, 95% CI 0.26–1.23), PANSS positive syndrome (P = 0.01, effect size = 0.85, 95% CI 0.34–1.34), negative syndrome (P = 0.01, effect size = 1.06, 95% CI 0.51–1.58), general psychopathology (P = 0.01, effect size = 1.78, 95% CI 1.08–2.47), GAF (P = 0.03, effect size = 0.51, 95% CI 0.05–0.96), LSP communication (P = 0.01, effect size = 0.67, 95% CI 0.19–1.14), nonturbulence (P = 0.02, effect size = 0.71, 95% CI 0.28–0.90), social contact (P = 0.01, effect size = 0.96, 95% CI 0.43–1.47), and total score (P = 0.01, effect size = 1.03, 95% CI 0.49–1.55).

**Table 2:** Comparison of each measure before and after JCORES and bridging sessions.

	Pre	Post	*p* value	Effect size (95% CI)
BACS				
Verbal memory	37.7 ± 11.0	44.9 ± 10.8	**0.01**	0.71 (0.22–1.18)
Working memory	18.2 ± 4.9	21.4 ± 4.9	**<0.01**	0.85 (0.34–1.35)
Motor speed	78.1 ± 12.6	86.7 ± 11.5	**<0.01**	1.03 (0.49–1.56)
Verbal fluency	38.7 ± 9.0	45.6 ± 11.7	**<0.01**	0.90 (0.38–1.40)
Attention and speed of information processing	51.8 ± 12.4	55.1 ± 11.9	0.06	0.44 (−0.02–0.88)
Executive function	16.6 ± 3.8	16.9 ± 3.7	0.60	0.13 (−0.34–0.55)
Composite score	39.9 ± 12.5	48.8 ± 11.8	**<0.01**	1.38 (0.97–1.00)
IFT				
Task‐dependent	5.5 ± 2.9	6.0 ± 2.4	0.40	0.19 (−0.25–0.62)
Task‐modified	2.2 ± 1.0	2.9 ± 1.9	**0.04**	0.58 (0.07–0.85)
Task‐independent	2.0 ± 1.8	2.2 ± 1.7	0.70	0.13 (−0.34–0.51)
DFT				
Task‐dependent	14.6 ± 19.0	15.5 ± 11.1	0.07	0.46 (0.00–0.75)
Task‐modified	3.7 ± 4.9	3.8 ± 3.5	0.58	0.19 (−0.40–0.67)
Task‐independent	6.7 ± 13.5	5.4 ± 9.0	0.89	−0.05 (−0.56–0.48)
m‐TTT				
Total score	8.7 ± 3.5	10.6 ± 3.6	**0.01**	0.75 (0.26–1.23)
PANSS				
Positive syndrome	20.0 ± 4.3	18.4 ± 4.5	**<0.01**	0.85 (0.34–1.34)
Negative syndrome	21.1 ± 3.3	18.2 ± 2.8	**<0.01**	1.06 (0.51–1.58)
General psychopathology	41.5 ± 6.2	37.7 ± 6.3	**<0.01**	1.78 (1.08–2.47)
GAF	52.8 ± 10.4	58.9 ± 12.3	**0.03**	0.51 (0.05–0.96)
LSP				
Communication	19.8 ± 3.1	21.4 ± 2.0	**0.01**	0.67 (0.19–1.14)
Self‐care	30.1 ± 3.1	30.8 ± 3.5	0.48	0.20 (−0.32–0.62)
Nonturbulence	44.6 ± 4.1	46.0 ± 2.5	**0.02**	0.71 (0.28–0.90)
Social contact	12.9 ± 4.0	15.7 ± 3.5	**<0.01**	0.96 (0.43–1.47)
Responsibility	18.8 ± 1.8	18.8 ± 2.4	0.50	0.28 (−0.46–0.79)
Total	126.1 ± 10.1	132.6 ± 9.0	**<0.01**	1.03 (0.49–1.55)

*Note*: Cohen's *d* effect size: very small effect size: *d* < 0.2; small effect size: 0.2 ≤ *d* < 0.5; medium effect size: 0.5 ≤ *d* < 0.8; large effect size: *d* ≥ 0.8. Bold values indicate statistically significant differences (*p* < 0.05).

Abbreviations: BACS, Brief Assessment of Cognition in Schizophrenia; CI, confidence interval; DFT, Design Fluency Test; GAF, Global Assessment of Function; IFT, Idea Fluency Test; JCORES, Japanese Cognitive Rehabilitation Program for Schizophrenia; LSP, Life Skills Profile; Modified TTT, Modified Tinkertoy Test; PANSS, Positive and Negative Syndrome Scale.

### Comparison between groups that transitioned to vocational activity and those that did not

The comparison between the vocational transition group and the non‐transition group 1 year after the JCORES intervention is shown in Table 3.

**Table 3:** Baseline characteristics of participants who engaged in employment or vocational activity and those who did not.

	1Y‐ET	1Y‐NET	*p* value	Effect size (95% CI)
Sex (male/female)	6/3	8/4	1.00	1.0 (0.16–6.3)
Age (years)	41.6 ± 7.7	40.1 ± 6.0	0.62	0.22 (−0.65–1.08)
Education history	13.4 ± 1.6	12.9 ± 1.9	0.51	0.29 (−0.58–1.16)
Total duration of employment (months)	81.0 ± 127.6	53.0 ± 63.8	0.51	0.19 (−51.7–120)
Duration of illness	15.8 ± 7.9	18.9 ± 8.6	0.40	0.38 (−0.58–1.16)
CP equivalent dose	554.3 ± 352.5	486.8 ± 244.2	0.61	0.23 (−0.65–1.08)
Anticholinergic use	2	4		
No anticholinergic use	7	8	0.66	0.57 (0.08–4.128)
Benzodiazepine use	3	5		
No benzodiazepine use	6	7	1.00	0.70 (0.12–4.23)
JART IQ	99.2 ± 9.6	98.4 ± 10.4	0.86	0.08 (−0.79–0.94)

Abbreviations: 1Y‐ET, 1‐year employment or vocational activity group (including both formal employment and participation in structured programs such as Type B continuous employment support); 1Y‐NET, 1‐year non‐transition group; CP, chlorpromazine; IQ, intelligence quotient; JART, Japanese Adult Reading Test.

The vocational transition group consisted of six males and three females, with an average age of 41.6 ± 7.7 years and an average education history of 13.4 ± 1.6 years. The total duration of prior employment was 81.0 ± 127.6 months. The average duration of illness was 15.8 ± 7.9 years, and the CP equivalent dose was 554.3 ± 352.5 mg. Among the participants, two were using anticholinergic medication, whereas seven were not. Three participants were using benzodiazepines, whereas six were not. The average JART IQ was 99.2 ± 9.6.

The nonvocational transition group consisted of eight males and four females, with an average age of 40.1 ± 6.0 years, an average education history of 12.9 ± 1.9 years, and the total duration of prior employment was 53.0 ± 63.8 months. The average duration of illness was 18.9 ± 8.6 years, and the CP equivalent dose was 486.8 ± 244.2 mg. Among the participants, four were using anticholinergic medication, while eight were not. Five participants were using benzodiazepines, whereas seven were not. The average JART IQ was 98.4 ± 10.4.

There were no significant differences between the groups regarding sex, age, educational history, total duration of employment, duration of illness, CP equivalent dose, anticholinergic medication, benzodiazepine use, and JART IQ.

Next, a comparison of changes in psychiatric symptoms, neuropsychological assessment scores, and community living skills before and after the intervention between the vocational transition and nontransition groups showed that the vocational transition group had a significantly greater reduction in negative symptoms (P = 0.03, effect size = 1.02, 95% CI −2.0–0.05) (Table 4).

**Table 4:** Comparison of baseline values and score changes between participants who engaged in employment or vocational activity and those who did not.

	Baseline			Change			
	1Y‐ET	1Y‐NET	*p* value	1Y‐ET	1Y‐NET	*p* value	Effect size
BACS							
Verbal memory	35.3 ± 11.6	39.5 ± 10.7	0.40	10.3 ± 5.8	4.8 ± 12.0	0.21	0.56 (−0.33 to 1.4)
Working memory	16.3 ± 5.2	19.7 ± 5.2	0.12	3.8 ± 5.1	2.8 ± 2.3	0.55	0.27 (−0.60 to 1.1)
Motor speed	75.3 ± 12.3	80.2 ± 13.0	0.40	7.6 ± 8.2	9.3 ± 8.6	0.64	0.21 (−1.1 to 0.66)
Verbal fluency	35.8 ± 7.9	40.8 ± 9.4	0.21	5.1 ± 10.0	8.3 ± 5.6	0.37	0.40 (−1.3 to 0.47)
Attention and speed of information processing	46.8 ± 11.3	55.6 ± 12.3	0.11	2.7 ± 9.6	3.8 ± 6.2	0.74	0.15 (−1.0 to 0.72)
Executive function	15.9 ± 4.3	17.2 ± 3.4	0.46	0.9 ± 3.1	₋0.3 ± 2.8	0.39	0.39 (−0.49 to 1.3)
Composite score	35.1 ± 10.5	43.5 ± 13.1	0.13	10.2 ± 7.9	7.9 ± 5.2	0.43	0.34 (−0.52 to 1.2)
IFT							
Task‐dependent	5.9 ± 3.1	5.3 ± 2.8	0.63	₋0.4 ± 2.3	1.2 ± 2.6	0.15	0.66 (−1.5 to 0.24)
Task‐modified	2.2 ± 1.0	2.2 ± 1.0	0.90	0.3 ± 1.0	0.9 ± 1.7	0.38	0.40 (−1.3 to 0.48)
Task‐independent	1.2 ± 1.1	2.6 ± 2.0	0.08	0.7 ± 1.7	₋0.2 ± 2.6	0.41	0.37 (−0.50 to 1.2)
DFT							
Task‐dependent	20.3 ± 27.6	10.3 ± 7.4	0.24	₋1.2 ± 16.1	1.2 ± 5.8	0.48	0.32 (−1.2 to 0.55)
Task‐modified	4.1 ± 6.8	3.4 ± 3.0	0.76	₋1.6 ± 2.4	1.3 ± 2.7	0.24	0.54 (−1.4 to 0.35)
Task‐independent	4.7 ± 12.1	8.3 ± 14.8	0.56	₋0.4 ± 2.3	1.2 ± 2.6	0.11	0.54 (−1.4 to 0.35)
m‐TTT							
Total score	8.1 ± 3.9	9.1 ± 3.3	0.54	1.4 ± 2.8	2.3 ± 2.5	0.45	0.34 (−1.2 to 0.54)
PANSS							
Positive syndrome	19.4 ± 3.7	20.5 ± 4.9	0.59	₋2.6 ± 2.5	₋0.9 ± 1.0	0.05	0.93 (−1.8 to 0.01)
Negative syndrome	22.1 ± 2.9	20.1 ± 3.0	0.08	−3.2 ± 2.2	₋1.3 ± 1.5	**0.03**	1.02 (−2.0 to 0.05)
General psychopathology	41.2 ± 7.4	41.8 ± 5.3	0.85	₋4.2 ± 2.1	₋3.5 ± 2.2	0.46	0.34 (−1.2 to 0.54)
GAF	50.9 ± 12.3	54.2 ± 9.0	0.49	5.8 ± 16.4	6.4 ± 8.3	0.90	0.05 (−0.92 to 0.81)
LSP							
Communication	18.8 ± 2.7	20.5 ± 3.2	0.21	1.6 ± 2.9	1.8 ± 2.2	0.86	0.08 (−0.94 to 0.79)
Self‐care	30.1 ± 3.6	30.2 ± 2.8	0.97	₋1.4 ± 3.7	2.1 ± 2.9	0.10	0.62 (−2.0 to 0.16)
Nonturbulence	44 ± 4.4	45 ± 4.1	0.60	1.9 ± 3.1	1.0 ± 2.1	0.45	0.34 (−0.5 to 1.2)
Social contact	11.7 ± 4.1	13.8 ± 3.8	0.24	3.7 ± 2.3	2.2 ± 3.3	0.26	0.34 (−0.5 to 1.2)
Responsibility	18.7 ± 1.2	18.9 ± 2.1	0.76	₋0.4 ± 3.1	0.3 ± 0.9	0.42	0.36 (−1.2 to 0.51)
Total	123.2 ± 10.0	128.3 ± 10.1	0.26	5.2 ± 6.5	7.4 ± 6.3	0.44	0.35 (−1.2 to 0.53)

*Note*: Cohen's *d* effect size: very small effect size, *d* < 0.2; small effect size, 0.2 ≤ *d* < 0.5; medium effect size, 0.5 ≤ *d* < 0.8; large effect size, *d* ≥ 0.8. Bold value indicates statistically significant differences (*p* < 0.05).

Abbreviations: 1Y‐ET, 1‐year employment or vocational activity group (*n* = 9, four participants in formal employment and five in vocational activity such as Type B support); 1Y‐NET, 1‐year non‐transition group; BACS, Brief Assessment of Cognition in Schizophrenia; IFT, Idea Fluency Test; DFT, Design Fluency Test; m‐TTT, modified Tinkertoy Test; PANSS, Positive and Negative Syndrome Scale; GAF, Global Assessment of Functioning; LSP, Life Skills Profile.

In the logistic regression analysis with vocational transition (transitioned vs. not transitioned) as the dependent variable and change in positive and negative symptoms as independent variables, the change in negative symptoms (odds ratio = 1.96, 95% CI [1.03–3.72], P = 0.01) was identified as a factor influencing vocational transition, with a discrimination accuracy of 76.2% (Table 5).

**Table 5:** Analysis of factors associated with employment or vocational activity transition.

Variable	Odds ratio	95% CI	*p* value
Change in negative symptoms (pre–postintervention)	1.961	1.03–3.72	0.01

*Note*: Employment or vocational activity transition was defined as engagement in either formal employment under a labor contract or participation in structured vocational programs (e.g., Type B continuous employment support) for at least 3 months during the 1‐year follow‐up period.

Independent variables were selected based on effect sizes (Cohen's *d* ≥ 0.8) from group comparisons. A stepwise logistic regression method was used.

Input variables: change in PANSS positive symptoms (pre–post), and change in PANSS negative symptoms (pre–post).

Discrimination accuracy: 76.2%.

These variables were selected based on effect size criteria (Cohen's *d* > 0.8) observed in the prior group comparison between the vocational transition and non‐transition groups. Only the changes in PANSS positive and negative symptom scores met this threshold, and were included to minimize the risk of overfitting given the small sample size.

## DISCUSSION

The vocational transition rate of 42.8% in this study suggests that CRT may support employment or participation in structured vocational activities among individuals with schizophrenia. Previous studies have reported vocational transition rates ranging from 4% to 50%, depending on factors such as work history, symptom severity, and vocational support.^14^ Compared to these rates, our findings indicate that CRT may contribute to improved vocational outcomes.

We also reported a vocational transition rate of 1.7% among non‐CRT participants in the same psychiatric daycare facility for context. While the study did not directly compare CRT and non‐CRT groups, the low rate among the general population highlights structural limitations in psychiatric rehabilitation, where access to goal‐oriented vocational support remains limited. Although CRT participants were self‐selected based on interest in vocational goals, the contrast suggests a need for broader implementation of targeted interventions.

A key finding was the association between reduced negative symptoms and vocational transition. Logistic regression showed that improvement in negative symptoms predicted vocational transition (odds ratio 1.961, 95% CI 1.03–3.72, P = 0.01), with a discrimination accuracy of 76.2%. This aligns with prior findings that negative symptoms hinder recovery and employment.^40^, ^41^ However, it remains unclear whether the observed reduction reflects changes in primary negative symptoms (deficit syndrome) or secondary ones (e.g., depression, medication side effects).

The direction of this association should be interpreted cautiously. It is possible that individuals more motivated or already preparing for vocational engagement showed greater symptom reduction. Alternatively, those with more modifiable secondary symptoms may have benefited more from CRT. As this study did not perform an item‐level analysis of PANSS‐negative symptoms, future research should examine specific items to determine which are most influenced by CRT and related to vocational outcomes.

Secondary symptoms are often more modifiable, suggesting that improvements observed here may stem from increased motivation, social engagement, or medication adjustments rather than direct effects of CRT on primary negative symptoms. Future work should distinguish primary from secondary symptoms to better understand their respective roles.

Consistent with previous studies, the CRT program (JCORES) improved PANSS negative and general psychopathology scores, BACS verbal memory, motor speed, and composite scores.^20^, ^24^ Meta‐analyses show moderate cognitive improvements from CRT and small‐to‐moderate effects on negative symptoms.^42^, ^43^, ^44^, ^45^ These results suggest CRT, especially when paired with interventions for secondary symptoms, may enhance vocational outcomes by addressing both cognitive and clinical impairments. While CRT alone may not directly reduce negative symptoms, its cognitive and social components may help alleviate secondary symptoms and promote functional gains. Further studies should test whether CRT combined with symptom‐targeted approaches improves vocational results.

This study demonstrated large effect sizes in psychiatric and cognitive domains, except for attention, processing speed, and executive function. Idea Tm and m‐TTT showed medium effect sizes, while life skill measures like communication and non‐turbulence were moderate; social contact and total scores were large. These results support CRT's utility across multiple functional domains, though they should be interpreted with caution due to methodological limitations.

CRT also improved verbal divergent thinking (IFT) but not nonverbal creativity (DFT). This indicates CRT's strength in enhancing verbal cognitive flexibility, but limited impact on visuospatial ideation. Verbal improvement likely stems from CRT's focus on executive function and strategic thinking. In contrast, DFT involves visuospatial and motor functions, not directly addressed in CRT. Future studies should test whether incorporating visuospatial components into CRT improves broader creativity and applicability.

Though negative symptom reduction was associated with vocational transition, other factors such as age,^19^ premorbid IQ,^20^ medication dose,^21^ and baseline cognitive function^23^ were not significantly different between groups. The use of anticholinergic or benzodiazepine medications also showed no difference. These findings emphasize the unique role of negative symptom improvement. Still, vocational outcomes are multifaceted, and future research should explore influences like workplace integration programs, social networks, and individual motivation.

To maximize CRT's vocational impact, complementary strategies such as goal‐setting, structured feedback, and real‐world role‐play may help transfer cognitive gains to practical outcomes. Techniques from divergent thinking training may also reduce secondary symptoms like apathy.^45^ Prior studies suggest that applying CRT skills in real‐life settings is more effective than cognitive drills alone.^46^ A meta‐analysis also found CRT led by trained therapists produced stronger results than unguided programs.^47^ These insights highlight the importance of structured, interactive CRT delivery.

One limitation is the potential for practice effects in cognitive assessments. We used BACS alternate forms (A and B) to reduce this, though such effects may not have been eliminated. Also, improvements could reflect procedural learning rather than true cognitive gains. Including control tasks in future studies would help isolate CRT effects.

This study's single‐arm pre‐post design without a control group is another limitation. Without a comparator, it's difficult to attribute observed improvements solely to CRT. Randomized controlled trials are needed to confirm CRT's true effect size.

Another limitation is the lack of direct assessment of vocational motivation. All participants voluntarily enrolled in CRT, suggesting some degree of interest, but differences in intrinsic motivation may have influenced outcomes.^48^ Future studies should measure motivation objectively through scales or interviews to better understand its influence.

Finally, all assessments were unblinded. PANSS ratings were performed by primary physicians, introducing potential bias. Blinded or independent raters in future trials could improve data reliability. Moreover, we used composite PANSS scores, limiting item‐specific insights. Some individual items, such as attention or depression, may be more closely linked to vocational function.^49^ Future studies should perform item‐level analyses to clarify these relationships.

Despite these limitations, this study offers important insights into CRT's relationship with vocational outcomes in schizophrenia. By identifying key cognitive and clinical predictors of vocational transition, the findings support CRT as a critical element of psychiatric rehabilitation. Further work should include controlled trials, motivation assessment, and detailed symptom evaluation to clarify mechanisms and enhance CRT effectiveness.

## CONCLUSION

This study highlights the potential role of negative symptom reduction in facilitating vocational transition. However, CRT is primarily designed to enhance cognitive functions, and its direct impact on negative symptoms remains limited. While our findings suggest that CRT may improve overall vocational and functional outcomes, previous studies have consistently reported small effect sizes for its impact on negative symptoms, therefore these results should not be interpreted as evidence that CRT enhances overall vocational functioning.

Further research is needed to confirm these findings in larger controlled studies and to explore whether integrating interventions that specifically address negative symptoms could optimize CRT outcomes. Although this study provides meaningful insights, the findings should be interpreted with caution due to the limited sample size.

## AUTHOR CONTRIBUTIONS

Yasuhisa Nakamura conceptualized and designed the study, performed the analysis, and drafted the manuscript. Yasuhisa Nakamura is also the corresponding author responsible for communication with the journal during the manuscript submission, peer review, and publication process. Keito Shimada, Reiko Miyamoto, and Akihiro Koreki collected the data, assisted with data analysis, and contributed to the writing and editing of the manuscript. Sachiko Anamizu and Masaru Mimura contributed to the study design, provided critical revisions to the manuscript, and supervised the project.

## CONFLICT OF INTEREST STATEMENT

The last author (Masaru Mimura) is an Editorial Board member of *Psychiatry and Clinical Neurosciences Reports* and a co‐author of this article. To minimize bias, he was excluded from all editorial decision‐making related to the acceptance of this article for publication. Masaru Mimura declares no conflict of interest directly related to this work. The first author (Yasuhisa Nakamura) declares no conflicts of interest.

## ETHICS APPROVAL STATEMENT

The study protocol was reviewed and approved by the Ethics Committee of Kariya Hospital in Japan (Approval No. 29–5).

## PATIENT CONSENT STATEMENT

All the study participants provided written informed consent.

## CLINICAL TRIAL REGISTRATION

The trial was registered with the University Hospital Medical Information Network (UMIN) Clinical Trials Registry (UMIN000054690).

## Data Availability

Raw data were generated at Kariya Hospital. Data supporting the findings of this study are available upon request from the corresponding author, Yasuhisa Nakamura.
